# Case of Strangulated Hernia, in Which There Was Considerable Ambiguity, Owing to the Scrotum Having Been Bruised, and a Tobacco Glyster Administered Prior to the Patient's Admission

**Published:** 1826-07

**Authors:** 

**Affiliations:** the Middlesex Hospital.


*Case of Strangulated Hernia, in which there was considerable ambiguity, owing to the Scrotum having been bruised, and a Tobacco Glyster administered prior to the Patient's Admission.* Treated by Mr. Shaw, at the Middlesex Hospital.

James White, æt. twenty-five, a patrol.

Dec. 7th.—This man has a large tumor of the scrotum: it is black, as if it were severely bruised; it feels as if it contained a fluid, and is œdematous from the scrotum to the root of the penis. A harder tumor occupies the abdominal ring. He says that, last night, when running after a thief, he struck his belly against a post, and brought down a rupture. We must here notice, that these men attribute all that befals them to the performance of their duty; and that it is very unlikely that a blow upon the belly should force a portion of intestine down so as at once to form a large scrotal hernia. The belly is not at all distended, nor is there pain on pressure, except just in the neighbourhood of the ring; but he is in an extraordinary state of suffering and exhaustion. His pulse is weak and fluttering; his countenance very pale; his skin cold and clammy; and he is sick: in short, he exhibits the symptoms which threaten death, in the last stage of strangulated hernia.

On inquiry, it is found that he has had a clyster of tobacco infusion, which throws some light upon the symptoms. We also learn that attempts have been made to reduce the gut: (he afterwards admitted that he had used very forcible means himself.) As the symptoms seemed to be referrible to the effect of the tobacco, and the blackness to the attempts made to reduce the hernia, it was resolved by the surgeons to meet again.

Before their next visit (which was made at four o'clock,) the man's mother had arrived, who stated that her son, when a child, had a "windy rupture;" and the patient, having now rallied a little, said that his rupture often came down, but that he could return it at night. The taxis had been repeatedly tried in the morning by the house-surgeon, but without success, and principally because the patient was in such a state of apathy that he always turned round on his belly, begging to be let alone; so that it was almost impossible to do any thing. As the symptoms have been gradually improving, the attempt to reduce the tumor by the hand was again made.

Four o'clock.—The tumor, when now examined, though large, gives the idea of being formed by a very thin sac, such as might be expected in a congenital hernia. On using the proper efforts at reduction, there appears a sort of crepitus, as if a portion of air were escaping from the hernia, which gives promise of final success; and the hope is further increased by the circumstance, that this seeming emptying of the hernia gives rise to tormina of the bowels; but still the patient, is in a very peculiar state, and apparently writhing under the pain, will not submit to further attempts. He again turns upon his belly, and will not keep himself in the right position, or submit to further pressure. The surgeons were thus obliged to leave him, and ordered the following pill:

℞. Colocynth. gr. v. Calomel, gr. iii. Opii, gr. ss. M. fiat pilula.

About an hour and a half after he took this pill, he had an injection of salt and gruel; and in the evening the pill was repeated.

Eight o'clock.—The attempts at reduction have been renewed. The tumor is diminished, and softer; he has had motions. He says he thinks he can now reduce it himself, which he promises at least to attempt. The symptoms are altogether better, and he is relieved from that state of excessive lowness and sickness which seemed to urge the necessity of an immediate operation. All idea of operating is given up.

Dec. 8th.—His condition improves; the blackness of the scrotum is changed to a red colour, and is now dissipating: part of the tumor, however, is still down. He has had motions.

Dec. 9th.—The intestine is now reduced, and the scrotum quite empty.

In the remarks made on this case, the perplexing nature of the symptoms was pointed out. Although they were very similar to those of a patient about to die of strangulated intestine, the tumor seemed likely to be reduced: its character, however, could not be satisfactorily ascertained, in consequence of the patient's unwillingness to submit to the taxis. This necessarily made the case more obscure. The scrotum was black, but this formed no indication of the state of the parts within. The principal obscurity in the symptoms arose from the effects produced by the tobacco glyster.

---

